# Therapeutic role of gut microbiota in lung injury-related cognitive impairment

**DOI:** 10.3389/fnut.2024.1521214

**Published:** 2025-02-13

**Authors:** Yanxia Cheng, Guangtao Hu, Lin Deng, Yalan Zan, Xia Chen

**Affiliations:** Department of Pediatrics, Child and Adolescent Psychiatric Center of Jiangbei Campus, The First Affiliated Hospital of Army Medical University (Army 958th Hospital), Chongqing, China

**Keywords:** gut microbiota, lung injury-related cognitive impairment, “triple-hit” hypothesis, “gut-lung-brain axis”, “lung-brain axis”, “lung-gut axis”, “gut-brain axis”

## Abstract

Lung injury can lead to specific neurocognitive dysfunction, and the “triple-hit” phenomenon may be the key theoretical mechanism for the progressive impairment of lung injury-related cognitive impairment. The lung and brain can communicate biologically through immune regulation pathway, hypoxic pathway, neural circuit, mitochondrial dysfunction, and microbial influence, which is called the “lung-brain axis.” The gut microbiota is a highly complex community of microorganisms that reside in the gut and communicate with the lung via the “gut-lung axis.” The dysregulation of gut microbiota may lead to the migration of pathogenic bacteria to the lung, and directly or indirectly regulate the lung immune response through their metabolites, which may cause or aggravate lung injury. The gut microbiota and the brain interact through the “gut-brain axis.” The gut microbiota can influence and regulate cognitive function and behavior of the brain through neural pathway mechanisms, immune regulation pathway and hypothalamic–pituitary–adrenal (HPA) axis regulation. Based on the gut microbiota regulation mechanism of the “gut-lung axis” and “gut-brain axis,” combined with the mechanisms of cognitive impairment caused by lung injury, we proposed the “triple-hit” hypothesis. It states that the pathophysiological changes of lung injury trigger a series of events such as immune disorder, inflammatory responses, and microbiota changes, which activate the “lung-gut axis,” thus forming a “triple-hit” that leads to the development or deterioration of cognitive impairment. This hypothesis provides a more comprehensive framework for studying and understanding brain dysfunction in the context of lung injury. This review proposes the existence of an interactive tandem network for information exchange among the gut, lung, and brain, referred to as the “gut-lung-brain axis.” It further explores the potential mechanism of lung injury-related cognitive impairment caused by multiple interactions of gut microbiota in the “gut-lung-brain axis.” We found that there are many numerous pathophysiological factors that influence the interaction within the “gut-lung-brain axis.” The impact of gut microbiota on cognitive functions related to lung injury may be mediated through mechanisms such as the “triple-hit” hypothesis, direct translocation of microbes and their metabolites, hypoxic pathway, immune modulation, vagal nerve activity, and the HPA axis regulation, among others. As the research deepens, based on the “triple-hit” hypothesis of lung injury, it is further discovered that gut microbial therapy can significantly change the pathogenesis of the inflammatory process on the “gut-lung-brain axis.” It can also relieve lung injury and therapeutically modulate brain function and behavior. This perspective provides a new idea for the follow-up treatment of lung injury-related cognitive impairment caused by dysregulation of gut microbiota.

## 1 Introduction

Lung injury leads to a decline in lung function and adversely affects the central nervous system (CNS). Cognitive impairment, a common complication of lung injury, severely impacts patients’ quality of life and social function (1). Cognition involves a series of higher cerebral cortex activities like perception, learning, execution, thinking, and judgment (2). Such impairment can vary from mild cognitive impairment (MCI) to Alzheimer’s disease. The possible mechanisms of cognitive dysfunction in patients with lung injury include immune regulation pathway, hypoxic pathway, neural circuit, mitochondrial dysfunction, and microbial influence. The gut microbiota is a complex microbial community in the gut, sharing a beneficial symbiotic bond with the host (3). Its dysregulation can cause or exacerbate lung injury via the “gut-lung axis.” Studies on gut microbiota homeostasis, such as in bacterial infections, administration of antimicrobial peptides or probiotics, or in germ-free mice, have demonstrated that modulating the gut microbiota can influence behavior and cognition (4). In recent years, we have realized that the “gut-brain axis” and “lung-brain axis” are crucial for cognitive function. As research has progressed, emerging evidence from clinical trials and animal models suggests the existence of an interactive tandem network of information exchange between the gut, lungs, and brain known as the “gut-lung-brain axis.” The pathways along the “gut-lung-brain axis” synergistically regulate immune responses, endocrine function, brain cognition and behavior. With the in-depth study of gut microbiota and their metabolites from the “gut-lung-brain axis,” it has known that abnormal changes in the host’s gut microbiota composition and abundance can cause cognitive impairment via this axis. In this review, we analyzed and explored the potential mechanisms of gut microbiota’s multiple interactions in the “gut-lung-brain axis” causing lung injury-related cognitive impairment. We also looked into the potential clinical value of gut microbiota in treating such impairment.

## 2 Lung injury-related cognitive impairment

Acute Lung Injury (ALI) and its severe form Acute Respiratory Distress Syndrome (ARDS) are clinical disorders that result in progressive damage to alveolar epithelial cells and capillary endothelial cells with a poor prognosis. Although a large number of studies and therapeutic trials have been conducted, measures to effectively prevent or treat lung injury remain limited. The mortality rate of acute lung injury is as high as 40–50% (5). Many studies have shown that ALI/ARDS survivors may experience cognitive impairment, including impaired memory, executive function, attention and decreased cognitive processing speed. These impairments usually persist during the acute inflammatory phase and after discharge from hospital, which in turn affects quality of life (6, 7). Cognitive impairment manifests itself as cognitive and behavioral deficits, often with inflammation and structural changes in the brain. Lung injuries that can lead to cognitive impairment include, but are not limited to, ventilator-induced lung injury (VILI), injuries caused by lung infections and inflammation, chronic obstructive pulmonary disease (COPD), and asthma (8, 9). VILI mostly shows inflammatory cell infiltration and cytokine release and can be classified into four mechanisms: pneumatic pressure injury, volumetric injury, injuries caused by atelectasis, and biological injury. A number of studies have now shown that VILI may lead to cognitive impairment (10). Nearly one-third of mechanically ventilated patients have impaired performance on cognitive tests at 6 months, suggesting that cognitive impairment is common in VILI (11). Several studies have reported associations between lung infections and dementia, increased risk of cognitive impairment and structural brain differences (12, 13). CNS dysfunction (e.g., encephalopathy, depression, anxiety, cognitive impairment) may occur during and after sepsis due to the systemic inflammatory response (14). The interaction between peripheral and central inflammation can lead to brain dysfunction and deformation of the nerve, however the mechanisms are unclear. Bonorino et al. established an experimental model of pulmonary sepsis in mice, which resulted in a systemic inflammatory response with brain damage, increased blood–brain barrier permeability and pro-inflammatory cytokine levels, neutrophil infiltration and microglia activation in the hippocampus (9). A meta-analysis found that *chlamydia pneumoniae* infection increased the incidence of Alzheimer’s disease (AD) more than fivefold (15). In addition, some pulmonary pathogens such as tubercle bacillus have been found to contribute indirectly to the development of AD using their own molecular mechanisms (16). Recent studies have shown that COVID-19 is also associated with cognitive impairment. Liu et al. showed that at least 20–45% of SARS-CoV-2 infected individuals develop a range of neurodegenerative disease as well as cognitive deficits (17, 18). These studies indicate a bidirectional communication between the lung and the brain, with cognitive deficits emerging that adversely affect recovery from pulmonary injury. For example, cognitive dysfunction may lead to a decrease in patients’ ability to self-manage, affecting adherence to treatment and thus the recovery process of lung injury. In addition, cognitive impairment may increase patients’ risk of hospital care and length of stay, further affecting the recovery of lung function. Considerable social and family cost burdens, impaired emotional well-being of caregivers, impaired social and occupational competence, and many other harms have been identified in patients discharged from hospitals with lung injury (11).

## 3 The direct mechanisms of lung injury-induced cognitive impairment

The pathological and physiological markers of acute lung injury are massive neutrophil infiltration, progressive damage to alveolar epithelial cells and capillary endothelial cells, and elevated levels of pulmonary pro-inflammatory cytokines and chemokines such as TNF-α and IL-1β (5). As stated, many studies have shown that lung injury can cause cognitive impairment. The biological communication pathway between lung and brain is termed the “lung-brain axis.” However, the complex mechanisms underlying the potential link between lung injury and cognitive impairment are still unclear. The proposed mechanisms mainly include the following. (1) Immune regulation pathway: Based on the suggestion that the immune defense of the brain barrier and systemic proinflammatory factors are associated with cognitive impairment, Sahu et al. established a ALI experimental mouse model (8). The experimental data confirmed that ALI would destroy the immune homeostasis of the lungs, infiltrate inflammatory cells into the lungs, and produce a large number of inflammatory cytokines and delay their regression, including TNF-α and IL-1β. Both TNF-α and IL-1β are associated with cognitive impairment. In addition to possible leakage from the lung, these inflammatory factors, mainly TNF-α and IL-1β, can be produced by activated leukocytes in the blood to induce systemic inflammation. Systemic inflammatory factors then destroy tight junctions (TJs), which are the main structure of the blood–brain barrier, resulting in increased permeability and structural disorder of the blood–brain barrier. Furthermore, the expression of these inflammatory factors is enhanced in the hippocampus, and finally leads to cognitive impairment in mice. In addition, systemic factors may help leukocyte extravasation by enhancing the expression of the adhesion molecule VCAM-1. This process subsequently leads to the expression of inflammatory mediators TNF-α and IL-1β, which in turn activate the brain’s own inflammatory response. Bonorino et al. established an experimental mouse model of pulmonary sepsis leading to systemic inflammatory responses, and confirmed that persistent neuroinflammation caused by high levels of inflammatory mediators (such as TNF-α, IL-1β, and IL-6) was considered a type of chronic brain dysfunction in sepsis survivors (9). Chen et al. found that ventilator-induced lung injury triggers a systemic inflammatory response. This may be associated with hippocampal structural damage and elevated levels of IL-1β, IL-6, and TNF-α in the hippocampus, which leads to blood–brain barrier rupture and cognitive impairment (19). (2) Hypoxic pathway: In addition to the aforementioned immune regulation pathway, the hypoxic pathway also plays an important role in the process of cognitive impairment induced by lung injury, and there may be a synergistic or progressive relationship between the two. Lung injury leads to the destruction of the integrity of the air-blood barrier, affecting the exchange of oxygen and carbon dioxide. Cerebral blood flow and metabolism disorders occur when hypoxia occurs (20), affecting the oxidative phosphorylation of hippocampal nerve cells (21). Hypoxia-induced hippocampal oxidative damage is associated with long-term brain dysfunction (22). In addition, neural transduction can induce neuronal apoptosis under hypoxic conditions of excitatory amino acids, energy depletion, and ion-related signals, and neuronal loss is an important pathological process of cognitive dysfunction (23). (3) Neural circuit: Hypoxemia/hypercapnia due to the hypoxia mechanism and stimuli like mitochondrial dysfunction, biological trauma, and alveolar stretching activate receptors in the airway and lung interstitium. These trigger upward neural signals that reach the brain through direct vagal connections or circulating factors (24, 25). In addition, lung injury can induce hippocampal inflammation by stimulating the vagus nerve with inflammatory factors (25). Recent studies have found a correlation between VILI and the imbalance of hippocampal neurotransmitter dopamine. Dopamine, as an anti-inflammatory substance, can significantly reduce lung tissue injury in rats by inhibiting the NOD-like receptor thermal protein domain associated protein 3 (NLRP3) signaling pathway. However, mechanical ventilation promotes the release of dopamine by increasing the expression of ATP and purinergic P2Y1G-protein-coupled receptor (P2Y1R) in strained lung epithelial cells, thereby aggravating brain injury (26). (4) Mitochondrial dysfunction: Mitochondria, as the “energy factories” of cells, also mediate the development of cognitive impairment induced by lung injury. Excessive mechanical ventilation can trigger mitochondrial damage and autophagy, leading to the release of mitochondrial DNA (mtDNA), which reduces brain ATP level and oxygen consumption, leading to cognitive impairment (27). (5) Microbial influence: In current microbiome medicine, some lung microbiota have been observed to have associated effects with specific neurological diseases. Recent research further suggests that the lung microbiota can influence brain autoimmunity and act as a bridge between the lung and brain. Hosang et al.’s study suggested that local inflammation and tissue damage in the lungs lead to dysbiosis of the lung microbiota, and the hilar microorganisms producing lipopolysaccharide (LPS) are reduced. LPS can enter the brain through the blood circulation through the blood–brain barrier and regulate the microglia in the brain, leading to a pro-inflammatory response (28).

Now many studies have pointed out that lung injury not only affects the brain through the “lung-brain axis,” but also the brain affects the lung through the “lung-brain axis.” For example, pulmonary complications such as neurogenic pulmonary edema (NPE), ARDS, and ventilator-associated pneumonia (VAP) may occur after traumatic brain injury (TBI) (29). It can be concluded that the lung and brain communicate with each other by triggering inflammatory factors or specific signaling pathways when subjected to traumatic stimulation, causing corresponding pathological changes. These studies demonstrate a bidirectional interaction of the “lung-brain axis.”

## 4 The changes of microbes in the lung and intestine during lung injury

Previously, the lower respiratory tract was considered “sterile,” but this perception is being challenged. Studies have shown that the bacterial communities in the lungs are similar to those in the oral cavity. Streptococcus, Prevotella, and Veillonella were the most common lung organisms. Bacteria of the phylum Firmicutes, Actinobacteria and Bacteroidetes are usually present in the healthy lung (30). The bacterial composition within the lower respiratory tract is determined by migration, elimination, and local growth conditions. In the case of respiratory diseases, changes in these factors can lead to the dysregulation of lung flora. In chronic inflammatory lung diseases, such as cystic fibrosis and chronic obstructive pulmonary disease, it has been confirmed that growth of the γ-Proteobacteria phylum, such as *Pseudomonas aeruginosa*, is significant. Factors that promote the expansion of *Pseudomonas aeruginosa* include the production of reactive oxygen species during inflammation, oxygen desaturation, and co-fermentation of mucin. The composition of the respiratory microbiota is also influenced by factors such as delivery mode and breastfeeding (31). A large number of studies have shown that patients have obvious lung dysbiosis in lung diseases (32). In patients with asthma, Haemophilus and Moraxella species were enriched. Haemophilus enriched in adults with asthma can induce the expression of Th17-related genes and is associated with asthma exacerbations (33). Compared with healthy subjects, the respiratory microbiota of asthmatic patients has lower bacterial diversity and higher richness, both of which are associated with asthma severity (34). Although most evidence suggests that gut-lung communication is primarily gut-to-lung, the opposite may also be the case. Chronic lung diseases, such as chronic obstructive pulmonary disease and cystic fibrosis, not only show the dysregulation of gut microbiota, but also show changes in the gastrointestinal tract, such as irritable bowel syndrome (30). It has been shown that influenza virus lung infection causes gut microbiota dysbiosis in mice, which results in reduced acetate levels. These reduced short-chain fatty acid (SCFA) levels have been associated with increased susceptibility to secondary pulmonary infections (35). On the other hand, the important role of the gut microbiota in acute viral and bacterial lung infections has been demonstrated in several mouse models after the reduction of gut bacteria by extensive antibiotic treatment. In mouse experiments, depletion of certain bacterial species in the gut microbiota of mice after antibiotic administration increased susceptibility not only to airway disease but also to viral infections in the lungs. Therefore, changes in the microbial composition of the gut microbiota can have a profound impact on host immune response and disease susceptibility (30).

## 5 Interactions of gut microbiota in lung injury

### 5.1 The gut microbiota influences lung diseases through the “gut-lung axis”

Pulmonary diseases refer to a group of diseases that affect airways and lung structures, leading to persistent breathing difficulties. Recently, studies have directly linked the onset and progression of lung diseases to the relative abundance of specific species in the gut microbiota (36). The gut microbiota has various essential functions in the human gut. It ferments nondigestible food components into absorbable metabolites, synthesizes essential vitamins, removes toxic compounds, defends against pathogens, strengthens the gut barrier, and stimulates and regulates the immune system. In addition to bacteria, the broad gut microbiota should also contain other key microorganisms, such as archaea, viruses, phages, and fungi (37). Gut microbiota can affect multiple organs, including the lung, and the interaction between gut microbes and the lung is known as the “gut-lung axis” (38). The disorder of gut microbiota can cause intestinal mucosal injury and inflammatory response, resulting in the migration of pathogenic bacteria to the lungs through the “gut-lung axis,” resulting in lung injury. The lung epithelium is comprised of pseudostratified ciliated columnar epithelial cells, whereas the gastrointestinal epithelium consists of either lamellar squamous epithelium or simple columnar epithelium (38). Despite these differences in structure, both types of epithelium originate from the endoderm and are exposed to external environments. The dynamics of the occurrence of the “gut-lung axis” may be attributed in part to the fact that the gastrointestinal tract has the same origin as the respiratory mucosa, as well as the similarities in physiological structure and function between the two (39). Gut microbiota can reach the lungs through lymph or blood circulation, triggering pulmonary inflammation and immune response. It can also affect the development and prognosis of lung diseases through immune regulation and metabolites of intestinal microbiota. Under pathological conditions, bacteria and endotoxin invading the intestinal mucosa can migrate to the lung injury site under the action of the “gut-lung axis.” The dysregulation of gut microbiota stimulates the gastrointestinal tract to release inflammatory mediators and cytokines. These substances can migrate to the lung through the capillary axis connecting the gut and the lung, mediating the infiltration of lung inflammatory cells and even causing lung injury. When the intestine is in a state of ischemia/reperfusion, succinic acid produced by gut microbiota will accumulate in the lungs and stimulate alveolar macrophages, leading to apoptosis of lung epithelial cells and severe pulmonary edema. Studies have shown that the interrelationship between the gut and lung is mainly mediated in two ways, including bacterial products in the circulation system and immune cells in the intestinal-pulmonary lymph nodes (40). The protein fraction of dead or viable bacteria produced in the gut can enter the systemic circulation along the mesenteric lymph nodes and then enter the pulmonary circulation. This stimulates pulmonary dendritic cells, macrophages, and T cells, leading to an influx of pulmonary neutrophils. Eventually, macrophage and neutrophil activation causes lung injury and inflammation. Another way in which the gut and lung interact is through the migration of T and B cells. In mesenteric lymph nodes, translocated bacteria and their products can initiate naive B cells and T cells, thereby activating the differentiation of B cells into plasma cells. However, plasma cells can reduce the production of anti-inflammatory molecules such as IL-10, leading to the initiation and differentiation of T cells. Some of these T cells will migrate out of gut-associated lymphoid tissues and reach the lung epithelium. Eventually, they activate local antigen presenting cells and T cells. The activation of these immune cells can clear pathogens in the lung, thereby reducing lung injury (38).

### 5.2 Effects of metabolites of the gut microbiota on lung injury

SCFA produced by intestinal symbiotic bacteria are the most abundant metabolites in the gut microbiota. SCFA, including butyrate, propionate, and acetate, are produced by the metabolism of dietary fiber by the gut microbiota. Their production is directly proportional to the content of dietary fiber (41). The underlying mechanism of SCFA action has been attributed to two major signaling pathways. The direct effects of SCFA on host immunity are mainly achieved through G protein-coupled receptors (GPCRs). These GPCRs are differentially expressed in a variety of cell types and tissues, including GPR43 (also known as free fatty acid receptor 2, FFAR2), GPR41 (FFAR3), and GPR109A (NIACR1) (30, 39). Butyrate in SCFA can inhibit lung type II lymphocytes and reduce airway hyperresponsiveness, thereby alleviating bronchial asthma. Another documented downstream signaling effect of SCFA is the regulation of immune responses by inhibiting histone deacetylase (HDAC) activity in various cell types (30, 39). Desaminotyrosine (DAT) also plays an important role in the “gut-lung axis.” DAT can modulate the pulmonary response by enhancing the type I interferon response. It also protected mice from hypersensitivity pneumonitis, asthma and influenza virus infection. In addition, the gut microbiota produces metabolites with both pro-inflammatory and anti-inflammatory potential, such as biogenic amines (including histamine), which have a profound impact on lung diseases through “gut-lung axis” interactions (33).

### 5.3 Effects of gut microbiota on the host immune system

The healthy gut microbiota plays an important role in the host’s local mucosal defense and pulmonary immune regulation. It can enhance immunotherapy through stimulator of interferon genes (STING) signaling (42). The gut microbiota’s bacterial components, LPS and peptidoglycan, bind to Toll-like receptors (TLRs) or NOD-like receptors (NLRs). These are expressed in gut cells as pattern recognition receptors (PRRs) to regulate immune responses (33). Crosstalk between the gut and lung occurs through the lymphatic and blood circulation systems and is critical for transmitting “immune information” between organs over long distances (39). This crosstalk may be mediated by pleiotropic metabolites synthesized by the gut microbiota, including folate, indole, secondary bile acids, neurotransmitters such as serotonin and γ-aminobutyric acid, and SCFA, which physiologically connect the gut and other organ systems (43). Regulatory T cells (Tregs) are a subset of T cells that play an important role in mediating the host immune response. Model studies have shown that Tregs are regulated by the gut microbiota and their metabolites. SCFA interact with host immunity by inhibiting histone deacetylation to increase the expression of transcription factor forkhead box protein 3 (FOXP3), supporting the expansion of Tregs and increasing IL-10 production (39). It has also been shown to enhance intestinal epithelial barrier function by increasing goblet cell differentiation and mucus production, as well as enhancing tight junction permeability. In addition, it can protects the gut from inflammation by enhancing the metabolism and differentiation of plasma B cells, thereby promoting intestinal IgA production. Gut microbiotic-derived metabolites can also signal through GPR43 and GPR109A on intestinal epithelial cells to induce NLRP3 inflammasome activation, an important cell survival and repair mechanism that protects against dextran sulfate sodium (DSS) -induced colitis (30). The effects of gut microbiota on peripheral immune cells as well as on the lungs are the basis for their promotion of lung homeostasis and immunity. An experimental study suggests that SCFA produced by the gut microbiota triggers its protective mechanisms against allergic airway diseases and respiratory infections. In addition, recent studies have suggested other mechanisms of action of SCFA fermented from gut microbiota. For example, it can affect the production of hematopoietic precursor cells in the bone marrow, thereby affecting the host immune system (30).

### 5.4 Antimicrobial peptides modify microbes to modulate lung injury

At present, the main approach to combat infection is still long-term aggressive antibiotic treatment, but this may lead to an increase in antibiotic resistance. In this case, antimicrobial peptides (AMPs) have received much attention because they may exhibit effective antibacterial effects against antibiotic-resistant strains without much toxicity (44). AMPs are a class of small-molecule peptides, typically consisting of 12–50 amino acid residues, with a wide range of antimicrobial activities. At present, more than 3,100 natural AMPs have been found (45). They are important effectors in the innate immune system and the first line of defense against pathogen infection. Therefore, antimicrobial peptides are also known as host defense peptides (HDPs) (46). In addition to antimicrobial activity, AMPs have anti-inflammatory, anti-biofilm, immunomodulatory and other activities (47). Previous studies have shown that AMPs have significant effects in animal and cellular models of acute lung injury, pulmonary fibrosis, and lung cancer (48). For the antibacterial mechanism of AMPs, early studies mainly focused on the destruction of bacterial membranes. AMPs also have intracellular activity and they are used to deliver drugs to target cells for therapy. The antibacterial mechanism of AMPs mainly includes two pore-forming models: the bucket-plate model and the ring-hole model. The current study suggests that the anti-inflammatory mechanism of AMPs may include the following two aspects: one is to preventing inflammation inducers from binding to their sensors, and the other is that it inhibits and regulates the expression of inflammation-related signaling pathways and transcription factors, and the anti-inflammatory mechanism of AMPs mainly targets the inflammation caused by LPS (47). When the body is stimulated or injured, natural AMPs are released and participate in immune regulation to maintain the stability of the internal environment. These AMPs are able to stimulate various molecules in the immune system, such as chemokines, in response to disease. In addition, studies have shown that AMPs have potential anti-*mycobacterium tuberculosis* (anti-MTB) and anti-cancer activities, so antimicrobial peptides can also be designed as anticancer peptides to inhibit the growth of bacteria and cancer cells (49). Both bacteria and cancer cells have a negatively charged surface, which can be disrupted by peptides because they are both cationic amphiphilic. A recent example is LL-37, an endogenous AMP derived from antimicrobial peptides. It is also an endogenous cationic peptide expressed in human immune cells and is active against both extracellular and intracellular tuberculosis bacilli. By disrupting the bacterial cell wall upon binding, it causes disintegration and rapid rupture within minutes (50).

## 6 The potential mechanisms of gut microbiota leading to lung injury-related cognitive impairment

Physiological studies on cognitive impairment have shown that cognitive function is affected by neuron loss, neurogenesis, synaptic plasticity decline, and neuroinflammation. These conditions may be related to the dysregulation of gut microbiota (51). There are complex intrinsic relationship between gut microbiota and cognitive function. While the specific mechanisms underlying this relationship are still in the early stages of exploration, it has been researched and observed that the dysregulation of gut microbiota may cause lung injury-related cognitive impairment through the “gut-lung-brain axis.” Studies have shown that the gut microbiota and the brain interact through the “gut-brain axis.” The gut microbiota can communicate with the CNS through the “bidirectional brain-gut pathways” such as neural pathway mechanisms, immune regulation pathway and HPA axis regulation, so that the gut microbiota can affect the brain and behavior (52). Therefore, the gut microbiota has been called the “second brain.” The role of the gut microbiota in the regulation of neurotransmitters is reflected in several ways: some gut microbiota encode genes for enzymes required for neurotransmitter synthesis, directly produce neuroactive compounds, or regulate the synthesis and release of neurotransmitters via enteroendocrine cells. In addition, neurotransmitters regulate the brain through three pathways: the one directly acts on the vagus nerve, the other impacts the enteric nervous system, and the remaining one involves crossing the blood–brain barrier (53). This regulatory mechanism reveals that the dysregulation of gut microbiota may trigger the disorder of neurotransmitter system, which in turn affects the cognitive function of the brain. There is increasing evidence that gut microbial antigens can initiate immune cells in gut-associated lymphoid tissue to reach the CNS and participate in immune surveillance (54). If the gut microbiota is in a state of dysbiosis, it may disrupt this interaction and lead to an inflammatory state. The inflammatory state can promote neuroinflammation and lead to cognitive decline. Therefore, the gut microbiota may influence the homeostasis of the CNS by regulating the immune system, or directly regulate the production of molecules and metabolites that affect the nervous and endocrine systems. The HPA axis is an important component of the neuroendocrine system and communicates through feedback interaction pathways. In this process, corticotropin releasing hormone (CRH) is secreted by the hypothalamus and acts as the pituitary gland. CRH stimulates the pituitary gland to produce adrenocorticotropin (ACTH), which ultimately targets the fascicular zone of the adrenal cortex to produce glucocorticoids (such as cortisol). Once these glucocorticoids are produced, they quickly enter the blood circulatory system and exert their extensive physiological effects. Due to the increased permeability of the intestinal mucosal barrier and the microbially driven pro-inflammatory state, the gut microbiota can activate the HPA axis and release glucocorticoids (cortisol and corticosterone) (55). Glucocorticoid receptors are widely expressed in the center, and appropriate concentrations of cortisol are essential for normal neurodevelopment and function, as well as cognitive processes such as learning and memory (56). Based on the direct mechanisms of lung injury-related cognitive impairment, and combined with the aforementioned mechanisms of gut microbiota regulation in “gut-lung axis” and “gut-brain axis,” it can be proposed that the gut microbiota has multiple interactions in the “gut-lung-brain axis” and leads to lung injury-related cognitive impairment. However, there are relatively few studies on the mechanism of “gut-lung-brain axis.” The known or predicted potential mechanisms mainly include the “triple-hit” hypothesis, direct translocation of microbes and their metabolites, hypoxic pathway, immune modulation, vagal nerve activity, and the HPA axis regulation.

### 6.1 The “triple-hit” hypothesis

**Figure 1:**
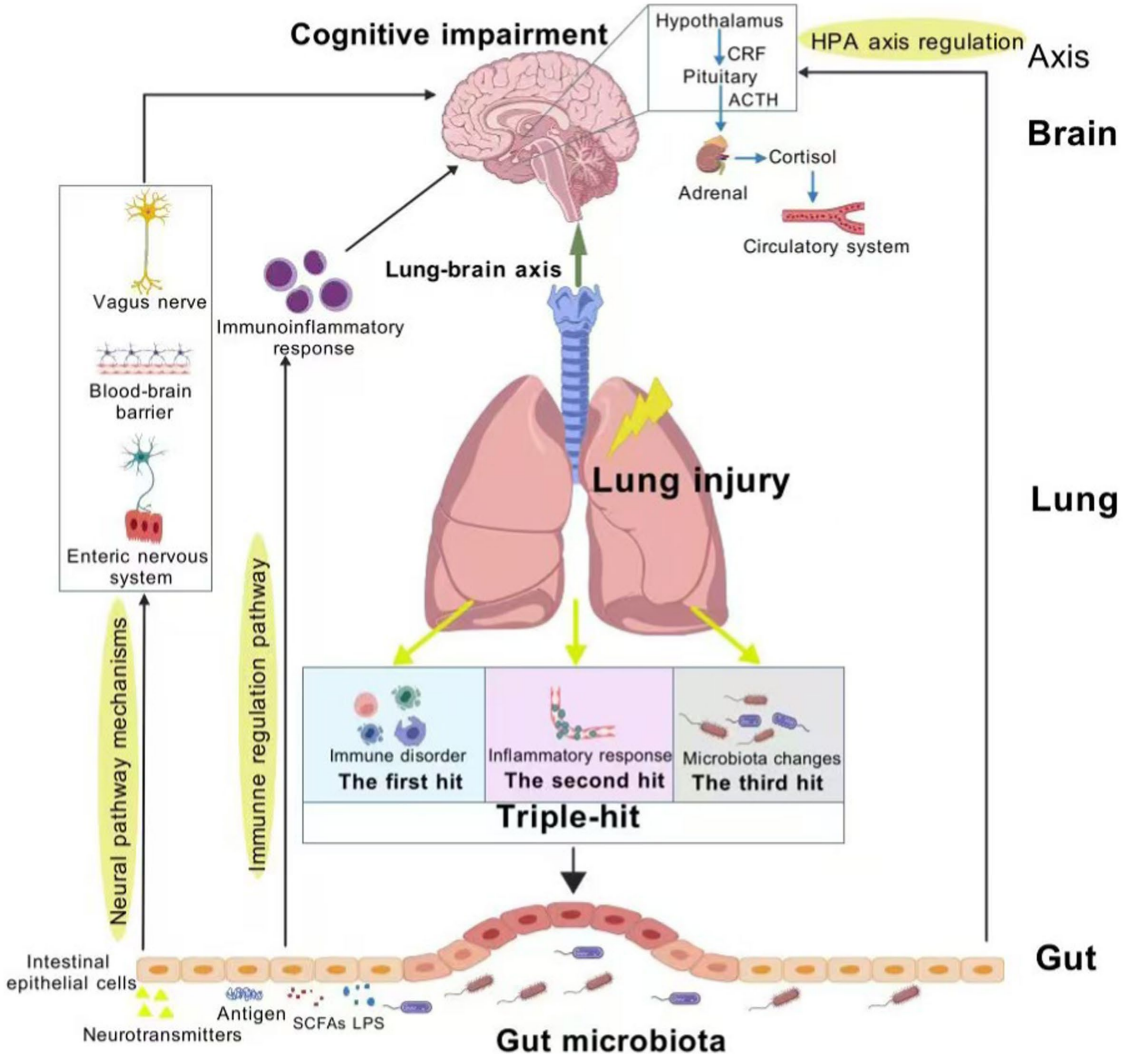
Schematic representation of the role of the “triple-hit” hypothesis in the gut-lung-brain axis. Figure created with BioGDP.com, licensed under CC BY-NC 4.0 (67).

The “triple-hit” hypothesis is constructed on the basis of immune disorder, inflammatory response, and microbiota changes in the lung tissues of patients with lung injury. Under the action of the “lung-gut axis,” the gut is affected by three effects. Specifically, the “triple-hit” results from the following pathophysiological processes. (1) Immune disorder: The lung and gut are organs in direct contact with the outside world, both of which have a typical mucosal structure composed of epithelium and lamina propria, and they share a mucosal immune system. When pathogens invade the lung or the respiratory mucosa is damaged, the local immune response mechanism of the lungs is triggered, and factors such as VEGF and TNF-α are produced. Consequently, the cadherin bond is destroyed, and the lung capillary endothelial injury leads to activated immune cells migrating to the gut, making the gut suffer the first hit. (2) Inflammatory response: Bacterial debris and products prompt the body to release circulating inflammatory cytokines and chemokines via the capillaries linking the lung and gut. This mediates intestinal inflammatory cell infiltration and mucosal damage, and may even extend to distant organs and systems, inducing systemic inflammatory response syndrome, which is the second hit (57–62). (3) Microbiota changes: Pathophysiological changes in lung structure and impaired mucous clearance mechanism lead to dysregulation of microbiota, and changes in the host’s microenvironment will further affect the colonization ability of the microbiota in the tissue, changing the composition of the microbiota, which forms a third hit (63). Changes in the composition of pulmonary microbiota can disrupt intestinal immune homeostasis, up-regulate the expression of inflammatory factors, and induce increased permeability of intestinal mucosal epithelium through the “lung-gut axis.” This leads to migration of bacterial flora and endotoxin. The synergistic effect of the three hits causes severe extrapulmonary effects that interfere with the ecological balance of the gut microbiota, thereby activating the gut-brain axis and causing the continuous progression of cognitive impairment (Figure 1). Lung injury is the initial step in the development of cognitive impairment, and the gut microbiota plays an important role in the progression of lung injury-related cognitive impairment. Thus, the “triple-hit” hypothesis can be used as a theoretical background for studying prevention or treatment strategies. These strategies will target the “gut-lung axis” and “gut-brain axis” to mitigate the risk of cognitive impairment, which is exacerbated by immunosuppression, systemic inflammation and microbiota translocation in susceptible patient populations.

### 6.2 Direct translocation of microorganisms and their metabolites

In recent years, it has been noted that a large number of enterogenous microorganisms are present in the lung microbiota of patients with lung injury. The original microbiome of the lung is replaced by enriched intestinal translocation bacteria such as Enterobacteriaceae, and is associated with the subsequent occurrence of ARDS (64). Dickson et al. reported that the bronchoalveolar lavage fluid specimens of ARDS patients were rich in intestinal microorganisms Pasteudiaceae and Enterobacteriaceae (62). Molecular techniques revealed the presence of diverse bacterial communities in the healthy lung, mainly including Prevotella, Veillonella, Streptococcus, and Fussobacterium. Due to the lack of nutrient substrates necessary for bacterial metabolism, the biomass of these microorganisms is very low (103–105 bacteria per gram of tissue) (65). However, changes in local pathophysiology and metabolites such as pH, oxygen concentration, free radical generation and nutrients in the alveoli during lung injury create a more favorable environment for the proliferation of potential pathogens (66). In this case, the surfactant layer loses activity and the mucociliary clearance function is impaired, which hinders the process of microbial clearance. During the stress response to lung injury, inflammatory mediators (IFN-γ, IL-6, TNF-α) destroy tight junctions and intestinal hypoperfusion and other factors will aggravate the increase of intestinal permeability and form “intestinal leakage.” Changes in the microbiota may modulate the pro-inflammatory response and exacerbate secondary brain injury. In a mouse model of LPS-induced severe pneumonia, the presence of bacteria in brain tissue was detected and the similarity in sample composition between the brain and lung microbiota was further established. This suggests that bacteria present in the brain may originate in the lungs. Behavioral experiments have also found that there are neurological abnormalities such as low spatial cognitive ability and low ability to recognize new things (68). Cotoia et al. analyzed the gut microbiota composition of 31 hospitalized patients with TBI and the changes in the lung microbiota of TBI patients with VAP, revealing for the first time that microbiome translocation may be the mechanism of similar bacterial content in the lung and gut of critically ill patients with brain injury (69). Luminal components from the small intestine, including gut microbes, pathogenic bacteria, LPS, and inflammatory molecules, reach the lungs via the portal circulation or mesenteric lymphatics, further contributing to alveolar inflammation and lung injury. Thus, microbial alterations in the lung can initially be attributed to the direct translocation of gut microbiota mediated by the circulatory system and the immune system (70). In addition, simultaneous impairment of the lung-blood barrier and the blood–brain barrier was observed during acute pneumonia, which also explains how gut microbiota invade the brain from the lungs (68).

### 6.3 Hypoxic pathway

Unlike the gastrointestinal tract, the environment in the alveolar space is not conducive to the growth of most bacteria. The nutrients available for bacterial growth and reproduction are very limited. Unlike the nutrient-rich intestinal cavity, the interior of the alveoli is only covered by a thin, lipid-rich surface active material on the lining of the epithelium. In cases of lung injury such as respiratory distress or pneumonia, the environmental conditions within the alveoli can change dramatically. For example, pulmonary edema or alveolar collapse can create a hypoxic or anaerobic environment, that creates more favorable conditions for the colonization of anaerobic microbiota (71). For patients with lung injury who need mechanical ventilation, they may continue to have a small amount of oropharyngeal microbiota. Coupled with impaired ciliary clearance of airway mucosa and disruption of the alveolar surface active substance layer, this predisposes to alveolar collapse. These alveolar environments that can cause lung injury progressively converge toward the intestinal tract (72). This could also account for why a significant proportion of the lung microbiota in critically ill patients suffering from lung injury originate from the gut. When patients with lung injury are in a state of hypoxia for a long time and reduced cerebral perfusion makes oxygen supply unable to meet the basic needs of brain metabolism, the brain tissue structure may be damaged, including neuronal injury and axonal degeneration (1). In this case, dysregulation of gut microbiota may exacerbate lung injury, which in turn may further disrupt the balance of the gut microbiota. This interaction can create a vicious cycle that continues to exacerbate cognitive impairment.

### 6.4 Immune regulation

The effect of lung injury on immune function is achieved by stimulation of pro-inflammatory mediators, and demyelination in the CNS is closely related to inflammatory response. The inflammatory response is coordinated by infiltrated T cells and endogenous glial cells (32). By establishing an autoimmune encephalomyelitis model, Murphy et al. Found that brain-derived T-cells with venous metastasis first locate in the lung, then proliferate and acquire the ability to migrate, and survive as memory cells (73). When these lung-resident memory T cells are activated by myelin basic protein, they migrate to the CNS and induce cognitive changes by activating microglia to induce inflammation in the CNS. In addition, the gut microbiota also has the ability to regulate the function of resident immune cells in the CNS, and its metabolites can not only regulate the immune response of the gastrointestinal tract, but also have an impact on distant organs such as the lung and brain (51). This finding underscores the importance of the immune system as a highly credible indirect communication pathway between gut microbiota, brain function, and lung injury-related cognitive impairment.

### 6.5 Vagal nerve activity

As one of 12 pairs of cranial nerves, the vagus nerve extends from the brain stem to the abdomen and connects the brain to all the vital organs of the organism through various organs, including the esophagus, heart, and lung (74). Most vagus nerves (80%) provide ascending sensory information, receiving signal input from thoracic tissues (such as the heart and lung) and abdominal tissues (such as the stomach and intestine). The increase of vagus nerve excitability can reduce the permeability of intestinal barrier, reduce the level of enteric-borne inflammatory substances in lung, and down-regulate the pulmonary inflammatory cascade, thus inhibiting the apoptosis of lung cells and alleviating lung tissue injury. The vagus nerve is the main conduit between the lung and the brain for normal breathing. Once stimulated, they can regulate lung and brain function by releasing acetylcholine or neuropeptides. There has been evidence that reduced pulmonary compliance can stimulate the vagus nerve, increase pulmonary vascular permeability, and activate sensory neurons that reach brain regions directly or through multisynaptic connections (75). In addition, cytokines released from damaged lung may also promote pro-inflammatory states in the brain, and these responses have been linked to long-term brain dysfunction. The vagus nerve is not only a neurocommunication pathway between the “lung-brain axis,” but also indirectly involved in the regulation of the immune system, neuropeptides and the composition of gut microbiota (76). Vagus nerve endings are widely distributed in the intestinal mucosa. The gut microbiota can communicate with the brain through the vagus nerve. This neural pathway may not directly interact with the gut microbiota, but indirectly perceive microbial signals through various bacterial metabolites and microbiota dependent immune mediators, thus affecting brain regions involved in cognition, emotion, somatosensory or eating behavior (51). In animal models, severing the vagus nerve can reverse behavioral changes triggered by gut microbiota transplantation (77). Thus, the effect of the gut microbiota on cognitive function on the “gut-lung-brain axis” may depend on the activation state of the vagus nerve.

### 6.6 The HPA axis regulation

Changes in the gut microbiota, whether due to diet, antibiotics, or other factors, can affect stress response, HPA axis activity, and overall cognitive health. HPA axis is a major stress response system, and its activation may change the pulmonary microbial environment, affect local pulmonary immune homeostasis and even systemic cell-mediated immunity. Once this immune function is compromised, its cascade affects the CNS. Studies have shown that the gut microbiota regulates the HPA axis throughout life. When the HPA axis is dysfunctional, the concentration of cortisol and pro-inflammatory molecules may increase. This increases intestinal barrier permeability and promotes the entry of Gram-negative bacteria into the blood circulation. Eventually, it induces chronic inflammation of the CNS, affecting emotional state, cognitive ability, and behavioral development (55, 78). The HPA axis is not only one of the important regulatory axes in organisms, but also participates in immune and inflammatory processes by regulating peripheral nervous system function and endocrine response. The regulation of the HPA axis by the gut microbiota is evidence of an association with the “gut-lung-brain axis,” suggesting that gut microbiota can activate the HPA axis and thus promote lung injury-related cognitive impairment.

## 7 The potential therapeutic effect of gut microbiota on lung injury-related cognitive impairment

The gut, lung and brain are a physiological whole that can achieve organic unity in function. Once the host or external environment changes, this physiological balance will be broken, which will lead to various diseases. This interaction may be mediated by a complex network of neural, endocrine, immune and metabolic signaling pathways. While there is considerable evidence supporting the effectiveness of probiotics in the treatment of cognitive impairment, the therapeutic effect of probiotics on lung injury-related cognitive impairment is unclear. It has been discussed that the dysregulation of gut microbiota is directly or indirectly related to lung injury-related cognitive impairment. The theory of “gut-brain axis” and “lung-gut axis” may explain the persistence of cognitive impairment even after the lung injury resolves. On the basis of the traditional symptomatic treatment for the protection of lung injury, the perspective should be shifted to the dysregulation of gut microbiota to implement more precise interventions. These measures aim to restore immune balance, reduce inflammatory response and re-establish the “lung-gut axis” ecological balance, while preventing changes in the lung and gut microbiota. Therefore, the adjustment of gut microbiota by means of probiotic or prebiotic supplement, fecal microbiota transplantation, and dietary intervention can improve lung injury-related cognitive impairment. They may competitively exclude intestinal pathogens by forming a physical barrier, blocking entry or generating antimicrobial properties, or stimulate the immune system by stimulating the production of anti-inflammatory cytokines. Moreover, they can maintain HPA axis function and intestinal barrier integrity by communicating the vagus nerve with the CNS and reducing inflammatory responses (79–81). In addition, the “triple-hit” hypothesis not only clarifies the relationship between lung injury and cognitive impairment, but the biological processes and mechanisms it reveals may become new therapeutic targets. Overall, therapies related to the “gut-lung-brain axis” for building a healthy gut microbiota will emerge as a novel safe potential treatment for the prevention and treatment of lung injury-related cognitive impairment.

## 8 Treatment strategies for microbiome therapy

### 8.1 Probiotic and prebiotic supplementation with individual differences

In summary, gut microbiota dysbiosis has a significant impact on the occurrence and development of lung injury, brain and cognitive impairment. Therefore, mechanisms that fully capture the “gut-lung-brain axis” communication will facilitate the development of microbiota-based therapies for lung injury-related cognitive impairment. Given the important role of gut microbiota, manipulation and regulation of gut microbiota is an effective therapeutic strategy for lung and brain diseases. This has been validated by an increasing number of clinical and experimental studies. These studies have tried prebiotics, probiotics and fecal microbiota transplantation (FMT) in patients with lung and brain diseases, aiming to shape the microbiota to improve the disease (82–85). Probiotics and prebiotics can significantly alter the pathogenesis of inflammatory processes by modulating the gut microbiota. This modulation involves enhanced proliferation of beneficial microorganisms and reduced presence of pathogenic microorganisms, aiming to introduce specific microbial strains to stimulate microbiota health-promoting pathways and increase the production of beneficial metabolites (86, 87). Although the definition of prebiotics is highly controversial, it most commonly refers to dietary carbohydrates that are selectively fermented by the gut microbiota to modulate the composition of the microbiota, thereby conferring health benefits to the host (88). Prebiotics reach the site of action in the colon and are fermented by native beneficial saccharolytic microorganisms such as Bifidobacterium, and the end products of prebiotic fermentation include SCFA (89, 90). Probiotics can produce two different immunomodulatory effects on the host, which can induce proinflammatory or anti-inflammatory immune responses. Under the action of immune stimulation, the phagocytic activity of macrophages, dendritic cells, neutrophils and natural killer cells (NK) increases, the release of inflammatory cytokines increases, and the Th1/Th17 polarization in the intestinal mucosa increases (91). In the anti-inflammatory response, some probiotic strains can induce Tregs, interleukin-10 (IL-10) and TGF-*β*, and enhance IgA secretion and intestinal barrier function by regulating intestinal mucosal dendritic cells (92). In addition, prebiotics play a crucial role in the immunomodulatory properties of SCFA, the product of intestinal fermentation, which regulates the immune function of the gut and lung. For example, butyrate can act as a histone deacetylase inhibitor, suppress gene expression of proinflammatory cytokines, improve intestinal barrier function, induce regulatory T cells, and act as a signaling molecule of the “gut-brain axis.” Butyrate also mediates neuroimmune mechanisms, driving the inflammatory process of inflammation, as well as the integrity of the gut and blood–brain barrier (89, 90). SCFA produced by microorganisms may affect the expression of brain-derived neurotrophic factor (BDNF), thereby affecting various brain functions as well as the survival of existing neurons and the growth of new neurons.

Some Lactobacillus species can regulate the immune and nervous system through metabolites (93), and probiotic supplementation increases the level of fatty acids in the brain, which is important for brain function, learning, memory, and neurogenesis. In a randomized, double-blind, placebo-controlled clinical trial, *Bifidobacterium breve* improved cognitive function in healthy older adults with MCI, including repeatable battery for the assessment of neuropsychological status (RBANS) total score, immediate memory, visuospatial/structural score, and delayed memory (94). An increasing number of animal studies have shown that probiotic treatments (such as Lactobacillus and Bifidobacterium) can reduce intestinal permeability and inflammation, and increase the levels of neurotrophic factors (BDNF and GDNF), inhibit the activation of microglia and astrocytes, and reduce stress-induced HPA axis dysfunction (95). In a clinical trial, a multistrain synthetic probiotic (*Lactobacillus acidophilus*, *Lactobacillus rhamnosus*, Lactobacillus plant, *Bifidobacterium longum*, *Streptococcus thermophilus*, prebiotic inulin) was found to improve biomarkers of oxidative, ultimately improving depressive symptoms, cognitive impairment, quality of life, and well-being (96). A randomized, double-blind, placebo-controlled clinical trial found that patients with MCI generally had higher levels of Prevotella compared to cognitively intact subjects. Additionally, *Lactobacillus rhamnosus* and the prebiotic inulin can reduce the relative abundance of Prevotella and Dehalobacterium in the MCI group and improved cognitive function. It is proposed that these taxa may become the early key indicators of MCI, and the combination of probiotics, prebiotics and symbiotic bacteria can successfully improve cognitive impairment (97). These data suggest that probiotics and prebiotics act through specific strains and can be personalized to therapeutically regulate brain function and behavior through various communication pathways of the “gut-brain axis” and “gut-lung axis.”

### 8.2 Fecal microbiota transplantation

Fecal microbiota transplantation (FMT) refers to the transfer of the microbial ecosystem of a healthy donor to the gastrointestinal tract of a recipient, with the purpose of changing the intestinal microbiota of the recipient and treating diseases related to intestinal dysbiosis (98, 99). It is a broad and largely untargeted strategy for modulating the gut microbiota. FMT has made significant progress over the years, evolving from a relatively crude procedure of transferring fresh donor feces to a mainstream treatment option. This evolution has been made possible by the development of standardized FMT products with an increasing emphasis on their drug formulation, pharmacokinetics, pharmacodynamics, and toxicity (100). A key advantage of FMT is the ability to transfer favorable microorganisms and their complex systems that support the ecological stability of the microbiota (101). At present, many experimental models have investigated the application of FMT in neurodegenerative diseases. The findings indicate that FMT holds promise for correcting intestinal dysbiosis, reducing the incidence of neurodegenerative diseases, enhancing cognitive function, promoting beneficial microorganisms, and increasing advantageous metabolites. These effects contribute to restoring the integrity of both the intestinal barrier and the blood–brain barrier (102, 103). In animal experiments, fecal transplantation was administered directly by gavage to observe whether it could improve the pathophysiological state of animals (104). An important milestone for FMT has now been reached with the recent FDA approval of RBX2660 (Rebyota) and SER-109 (Vowst) for the prevention of recurrent *C. difficile* infection. These therapies are administered in capsule form, without the need for endoscopy, and with the use of an oral microbial therapy. The therapeutic potential of FMT in human diseases has been highlighted (105, 106). Chen et al. recruited 5 elderly patients with cognitive impairment and administered oral fecal bacteria capsules. They found that the cognitive scores of patients with MCI were improved or maintained after FMT compared with those before transplantation. However, the cognitive scores of patients with severe cognitive impairment did not deteriorate, and no adverse effects were reported during the study (107). The effectiveness of FMT may come from direct microbial action or indirect mechanisms on lung injury, such as the production of microbial-derived metabolites, secondary bile acids and SCFA, regulating the strong inflammatory response triggered by lung injury and reducing various cellular and soluble inflammatory mediators (108). These findings hold promise for the development of successful therapeutics to manage lung injury.

### 8.3 Limitations of microbiome therapy

Although microbiome therapy holds great promise for the treatment of cognitive impairment associated with lung injury, the main challenge is the lack of gut microbiota profile and the limited types of probiotics for treatment. The present researches on probiotics is mainly limited to Lactobacillus and Bifidobacterium, and there are few studies on other potentially beneficial microorganisms. Secondly, individual heterogeneity in the composition and/or function of the microbiota leads to individual differences in the therapeutic effect of probiotics, so it is necessary to develop personalized treatment approaches. In addition, probiotics are effective but suboptimal for disease treatment. This is mainly due to their susceptibility to gastric acid at low PH in the stomach and exposure to various digestive enzymes, resulting in inactivation and impaired biological activity of probiotics. The low adhesion ability and insufficient intestinal retention of probiotics greatly hinder their colonization in the gut (109). Prebiotics can affect the growth and activity of a variety of microorganisms, so it is difficult to predict and control the effect of prebiotics on the microbiota (110). In recent years, the concepts of probiotic and prebiotic derivatives, including synbiotic (a combination of probiotic and prebiotic) and postbiotic (bacterial metabolite, such as SCFA), have been gradually proposed and received widespread attention. However, relevant research is still in a relatively immature stage, and their application in the treatment of cognitive impairment needs further exploration. In the future, microbial diversity and availability research is expected to progress significantly, driven by cutting-edge technologies and improved methods. For example, integrating multi-omics technologies such as genomics, transcriptomics, proteomics, and metabolomics can comprehensively characterize the microbial composition and functional dynamics of the “gut-lung-brain axis.” This may enable a deeper understanding of the complex interactions among the three, microbial communities, and their impact on health and disease. Higher resolution gut and lung microbiota mapping facilitates the identification of rare, unexplored microbial species, as well as their interactions, functional roles and health implications (111). In addition, future studies may identify specific microbial signatures or metabolites as diagnostic or prognostic biomarkers for various lung and gut-related diseases (112), enabling early development of personalized treatment strategies for specific diseases with personalized microbiota therapies targeting the “gut-lung-brain axis.” In the future, the differences in supplementation time, administration mode, dose and follow-up time of specific probiotic strains should also be focused on (112), and several innovative strategies should be studied to improve the oral bioavailability and gut targeting ability of probiotics. Such as microcapsules, hydrogel capsules, nanoparticle capsules, nanoenzyme integration, nanocoating, mineral coating and photogenic probiotic systems (109, 113–116).

The application of FMT in treating neurodegenerative diseases is constrained by factors such as the extent of grafting, scalability challenges, and the absence of standardized protocols (100). The degree of microbial implantation is an important indicator of the clinical success of FMT (117). According to the results of two recent meta-analyzes, in recipients with unstable microbial communities, antibiotic pretreatment of the recipient has been recommended to implant a higher degree of donor microbes (118). Moreover, FMT should be administered in combination and repeatedly to promote strain and counteract the effects of the transplanted microbes on gut ecology and achieve long-term efficacy. This is an urgent need for standardized FMT products with good stability and repeatability in clinical practice (117). In addition, FMT has the potential risk of complications that could lead to serious or life-threatening infections, such as sepsis and exacerbation of inflammatory processes, and further studies are needed to ensure safety and efficacy in critically ill patients with ARDS (119). It has been shown that immunocompromised individuals undergoing FMT studies developed invasive infections with antibiotic-resistant *E. coli* strains, with one death. Therefore, the blood and stool testing of FMT donors, as well as the selection, preparation, storage conditions, and quality testing of fecal transplant preparations need to be carefully and intensively studied according to different diseases (120–121).

## 9 Conclusion

Based on the latest understanding of the “gut-lung-brain axis,” we propose the “triple-hit” hypothesis, which is incorporated into the concept of the “lung-gut axis” and “gut-brain axis,” and expands our current understanding of the pathogenesis of lung injury-related cognitive impairment. Considering factors such as the “triple-hit,” the direct and indirect effects of microorganisms and their metabolites, the hypoxia mechanism, immune regulation, vagus nerve, and HPA axis, the mechanism of “gut-lung-brain axis” crosstalk is elaborated in detail. It provides a more comprehensive framework for studying and understanding brain dysfunction in the context of lung injury. Microbial therapy significantly alters the inflammatory process of the “gut-lung-brain axis” by regulating the gut microbiota, therapeutically regulating brain function and behavior. Further research in this direction is expected to reveal new therapeutic strategies that may improve the prognosis of patients with lung injury-related cognitive impairment.

## References

[1] Wang T Mao L Wang J Li P Liu X Wu W. Influencing factors and exercise intervention of cognitive impairment in elderly patients with chronic obstructive pulmonary disease. Clin Interv Aging. (2020) 15:557–66. doi: 10.2147/cia.S245147, PMID: 32368022 PMC7183549

[2] Gudden J Arias Vasquez A Bloemendaal M. The effects of intermittent fasting on brain and cognitive function. Nutrients. (2021) 13:13. doi: 10.3390/nu13093166, PMID: 34579042 PMC8470960

[3] Cryan JF O'Riordan KJ Sandhu K Peterson V Dinan TG. The gut microbiome in neurological disorders. Lancet Neurol. (2020) 19:179–94. doi: 10.1016/s1474-4422(19)30356-4, PMID: 31753762

[4] Ziaka M Exadaktylos A. Pathophysiology of acute lung injury in patients with acute brain injury: the triple-hit hypothesis. Crit Care. (2024) 28:71. doi: 10.1186/s13054-024-04855-w, PMID: 38454447 PMC10918982

[5] Johnson ER Matthay MA. Acute lung injury: epidemiology, pathogenesis, and treatment. J Aerosol Med Pulm Drug Deliv. (2010) 23:243–52. doi: 10.1089/jamp.2009.0775, PMID: 20073554 PMC3133560

[6] Hopkins RO Weaver LK Chan KJ Orme JF Jr. Quality of life, emotional, and cognitive function following acute respiratory distress syndrome. J Int Neuropsychol Soc. (2004) 10:1005–17. doi: 10.1017/s135561770410711x, PMID: 15803563

[7] Pelosi P Rocco PR. The lung and the brain: a dangerous cross-talk. Crit Care. (2011) 15:168. doi: 10.1186/cc10259, PMID: 21722336 PMC3219008

[8] Sahu B Sandhir R Naura AS. Two hit induced acute lung injury impairs cognitive function in mice: a potential model to study cross talk between lung and brain. Brain Behav Immun. (2018) 73:633–42. doi: 10.1016/j.bbi.2018.07.013, PMID: 30026058

[9] Bonorino KC Iria Kraus S Henrique Cardoso Martins G Jorge Probst J Petry Moeke DM Dos Santos H. Lung-brain crosstalk: behavioral disorders and neuroinflammation in septic survivor mice. Brain Behav Immun. Health. (2024) 40:100823. doi: 10.1016/j.bbih.2024.100823, PMID: 39252983 PMC11381903

[10] Liu Y Cai X Fang R Peng S Luo W Du X. Future directions in ventilator-induced lung injury associated cognitive impairment: a new sight. Front Physiol. (2023) 14:1308252. doi: 10.3389/fphys.2023.1308252, PMID: 38164198 PMC10757930

[11] Patel BK Wolfe KS Patel SB Dugan KC Esbrook CL Pawlik AJ. Effect of early mobilisation on long-term cognitive impairment in critical illness in the USA: a randomised controlled trial. Lancet Respir Med. (2023) 11:563–72. doi: 10.1016/s2213-2600(22)00489-1, PMID: 36693400 PMC10238598

[12] Russ TC Kivimäki M Batty GD. Respiratory disease and lower pulmonary function as risk factors for dementia: a systematic review with Meta-analysis. Chest. (2020) 157:1538–58. doi: 10.1016/j.chest.2019.12.012, PMID: 31952950

[13] Wang J Song R Dove A Qi X Ma J Laukka EJ. Pulmonary function is associated with cognitive decline and structural brain differences. Alzheimers Dement. (2022) 18:1335–44. doi: 10.1002/alz.12479, PMID: 34590419 PMC10085529

[14] Denstaedt SJ Spencer-Segal JL Newstead M Laborc K Zeng X Standiford TJ. Persistent Neuroinflammation and brain-specific immune priming in a novel survival model of murine Pneumosepsis. Shock. (2020) 54:78–86. doi: 10.1097/shk.0000000000001435, PMID: 31415473 PMC7015772

[15] Maheshwari P Eslick GD. Bacterial infection and Alzheimer's disease: a meta-analysis. J Alzheimers Dis. (2015) 43:957–66. doi: 10.3233/jad-140621, PMID: 25182736

[16] Azzoni R Marsland BJ. The lung-brain axis: a new frontier in host-microbe interactions. Immunity. (2022) 55:589–91. doi: 10.1016/j.immuni.2022.03.015, PMID: 35417673

[17] Liu YH Wu QX Wang QH Zhang QF Tang Y Liu D. Tracking cognitive trajectories in older survivors of COVID-19 up to 2.5 years post-infection. Nat Aging. (2024) 4:1186–93. doi: 10.1038/s43587-024-00667-3, PMID: 38987646

[18] Lu J Li CJ Wang J Wang Y. Neuropathology and neuroinflammation in Alzheimer's disease via bidirectional lung-brain axis. Front Aging Neurosci. (2024) 16:1449575. doi: 10.3389/fnagi.2024.1449575, PMID: 39280699 PMC11392776

[19] Chen C Zhang Z Chen T Peng M Xu X Wang Y. Prolonged mechanical ventilation-induced neuroinflammation affects postoperative memory dysfunction in surgical mice. Crit Care. (2015) 19:159. doi: 10.1186/s13054-015-0882-0, PMID: 25887955 PMC4423516

[20] Hoiland RL Bain AR Rieger MG Bailey DM Ainslie PN. Hypoxemia, oxygen content, and the regulation of cerebral blood flow. Am J Physiol Regul Integr Comp Physiol. (2016) 310:R398–413. doi: 10.1152/ajpregu.00270.2015, PMID: 26676248 PMC4796739

[21] Bilotta F Giordano G Sergi PG Pugliese F. Harmful effects of mechanical ventilation on neurocognitive functions. Crit Care. (2019) 23:273. doi: 10.1186/s13054-019-2546-y, PMID: 31387627 PMC6685219

[22] Jacobs RA Aboouf MA Koester-Hegmann C Muttathukunnel P Laouafa S Arias-Reyes C. Erythropoietin promotes hippocampal mitochondrial function and enhances cognition in mice. Commun Biol. (2021) 4:938. doi: 10.1038/s42003-021-02465-8, PMID: 34354241 PMC8342552

[23] Banasiak KJ Xia Y Haddad GG. Mechanisms underlying hypoxia-induced neuronal apoptosis. Prog Neurobiol. (2000) 62:215–49. doi: 10.1016/s0301-0082(00)00011-3, PMID: 10840148

[24] Albaiceta GM Brochard L Dos Santos CC Fernández R Georgopoulos D Girard T. The central nervous system during lung injury and mechanical ventilation: a narrative review. Br J Anaesth. (2021) 127:648–59. doi: 10.1016/j.bja.2021.05.038, PMID: 34340836

[25] González-López A López-Alonso I Pickerodt PA von Haefen C Amado-Rodríguez L Reimann H. Lung Purinoceptor activation triggers ventilator-induced brain injury. Crit Care Med. (2019) 47:e911–8. doi: 10.1097/ccm.0000000000003977, PMID: 31567350 PMC6798751

[26] Wei W Sun Z He S Zhang W Chen S Cao YN. Mechanical ventilation induces lung and brain injury through ATP production, P2Y1 receptor activation and dopamine release. Bioengineered. (2022) 13:2346–59. doi: 10.1080/21655979.2021.2022269, PMID: 35034579 PMC8974168

[27] Manfredini A Constantino L Pinto MC Michels M Burger H Kist LW. Mitochondrial dysfunction is associated with long-term cognitive impairment in an animal sepsis model. Clin Sci (Lond). (2019) 133:1993–2004. doi: 10.1042/cs20190351, PMID: 31527095

[28] Hosang L Canals RC van der Flier FJ Hollensteiner J Daniel R Flügel A. The lung microbiome regulates brain autoimmunity. Nature. (2022) 603:138–44. doi: 10.1038/s41586-022-04427-4, PMID: 35197636

[29] Chen J Li T Ye C Zhong J Huang JD Ke Y. The lung microbiome: a new frontier for lung and brain disease. Int J Mol Sci. (2023) 24:24. doi: 10.3390/ijms24032170, PMID: 36768494 PMC9916971

[30] Dang AT Marsland BJ. Microbes, metabolites, and the gut-lung axis. Mucosal Immunol. (2019) 12:843–50. doi: 10.1038/s41385-019-0160-6, PMID: 30976087

[31] Stricker S Hain T Chao CM Rudloff S. Respiratory and intestinal microbiota in pediatric lung diseases-current evidence of the gut-lung Axis. Int J Mol Sci. (2022) 23:23. doi: 10.3390/ijms23126791, PMID: 35743234 PMC9224356

[32] Yang K He S Dong W. Gut microbiota and bronchopulmonary dysplasia. Pediatr Pulmonol. (2021) 56:2460–70. doi: 10.1002/ppul.25508 34077996

[33] Song XL Liang J Lin SZ Xie YW Ke CH Ao D. Gut-lung axis and asthma: a historical review on mechanism and future perspective. Clin Transl Allergy. (2024) 14:e12356. doi: 10.1002/clt2.12356, PMID: 38687096 PMC11060082

[34] Carr TF Alkatib R Kraft M. Microbiome in mechanisms of asthma. Clin Chest Med. (2019) 40:87–96. doi: 10.1016/j.ccm.2018.10.006, PMID: 30691719

[35] Sencio V Barthelemy A Tavares LP Machado MG Soulard D Cuinat C. Gut Dysbiosis during influenza contributes to pulmonary pneumococcal superinfection through altered short-chain fatty acid production. Cell Rep. (2020) 30:2934–47.e6. doi: 10.1016/j.celrep.2020.02.013, PMID: 32130898

[36] Sey EA Warris A. The gut-lung axis: the impact of the gut mycobiome on pulmonary diseases and infections. Oxf Open Immunol. (2024) 5:iqae008. doi: 10.1093/oxfimm/iqae008, PMID: 39193472 PMC11316619

[37] Lavelle A Hill C. Gut microbiome in health and disease: emerging diagnostic opportunities. Gastroenterol Clin N Am. (2019) 48:221–35. doi: 10.1016/j.gtc.2019.02.003 31046972

[38] Tan JY Tang YC Huang J. Gut microbiota and lung injury. Adv Exp Med Biol. (2020) 1238:55–72. doi: 10.1007/978-981-15-2385-4_5 32323180

[39] Espírito Santo C Caseiro C Martins MJ Monteiro R Brandão I. Gut microbiota, in the Halfway between nutrition and lung function. Nutrients. (2021) 13:13. doi: 10.3390/nu13051716, PMID: 34069415 PMC8159117

[40] Enaud R Prevel R Ciarlo E Beaufils F Wieërs G Guery B. The gut-lung Axis in health and respiratory diseases: a place for inter-organ and inter-kingdom Crosstalks. Front Cell Infect Microbiol. (2020) 10:9. doi: 10.3389/fcimb.2020.00009, PMID: 32140452 PMC7042389

[41] Silva C Rojony R Bermudez LE Danelishvili L. Short-chain fatty acids promote *Mycobacterium avium* subsp. hominissuis growth in nutrient-limited environments and influence susceptibility to antibiotics. Pathogens. (2020) 9:700. doi: 10.3390/pathogens9090700 32859077 PMC7559849

[42] Sun JY Yin TL Zhou J Xu J Lu XJ. Gut microbiome and cancer immunotherapy. J Cell Physiol. (2020) 235:4082–8. doi: 10.1002/jcp.29359, PMID: 31663125

[43] Cani PD. Human gut microbiome: hopes, threats and promises. Gut. (2018) 67:1716–25. doi: 10.1136/gutjnl-2018-316723, PMID: 29934437 PMC6109275

[44] Caselli L Rodrigues GR Franco OL Malmsten M. Pulmonary delivery systems for antimicrobial peptides. Crit Rev Biotechnol. (2024) 44:963–80. doi: 10.1080/07388551.2023.2254932, PMID: 37731338

[45] Lazzaro BP Zasloff M Rolff J. Antimicrobial peptides: application informed by evolution. Science. (2020) 368:368. doi: 10.1126/science.aau5480, PMID: 32355003 PMC8097767

[46] Zandsalimi F Talaei S Noormohammad Ahari M Aghamiri S Raee P Roshanzamiri S. Antimicrobial peptides: a promising strategy for lung cancer drug discovery? Expert Opin Drug Discov. (2020) 15:1343–54. doi: 10.1080/17460441.2020.1791080, PMID: 32749935

[47] Luo Y Song Y. Mechanism of antimicrobial peptides: antimicrobial, anti-inflammatory and Antibiofilm activities. Int J Mol Sci. (2021) 22:22. doi: 10.3390/ijms222111401, PMID: 34768832 PMC8584040

[48] Li S Li Y Liu Y Wu Y Wang Q Jin L. Therapeutic peptides for treatment of lung diseases: infection, fibrosis, and Cancer. Int J Mol Sci. (2023) 24:24. doi: 10.3390/ijms24108642, PMID: 37239989 PMC10218668

[49] Polinário G Primo L Rosa M Dett FHM Barbugli PA Roque-Borda CA. Antimicrobial peptides as drugs with double response against *Mycobacterium tuberculosis* coinfections in lung cancer. Front Microbiol. (2023) 14:1183247. doi: 10.3389/fmicb.2023.1183247, PMID: 37342560 PMC10277934

[50] Deshpande D Grieshober M Wondany F Gerbl F Noschka R Michaelis J. Super-resolution microscopy reveals a direct interaction of intracellular *Mycobacterium tuberculosis* with the antimicrobial peptide LL-37. Int J Mol Sci. (2020) 21:21. doi: 10.3390/ijms21186741, PMID: 32937921 PMC7555347

[51] Sun Y Ho CT Zhang Y Hong M Zhang X. Plant polysaccharides utilized by gut microbiota: new players in ameliorating cognitive impairment. J Tradit Complement Med. (2023) 13:128–34. doi: 10.1016/j.jtcme.2022.01.003, PMID: 36970456 PMC10037067

[52] Peterson CT. Dysfunction of the microbiota-gut-brain Axis in neurodegenerative disease: the promise of therapeutic modulation with prebiotics, medicinal herbs, probiotics, and Synbiotics. J Evid Based Integr Med. (2020) 25:2515690X2095722. doi: 10.1177/2515690x20957225, PMID: 33092396 PMC7586271

[53] He Y Wang K Su N Yuan C Zhang N Hu X. Microbiota-gut-brain axis in health and neurological disease: interactions between gut microbiota and the nervous system. J Cell Mol Med. (2024) 28:e70099. doi: 10.1111/jcmm.70099, PMID: 39300699 PMC11412916

[54] Yang Y Xu Z Guo J Xiong Z Hu B. Exploring the gut microbiome-postoperative cognitive dysfunction connection: mechanisms, clinical implications, and future directions. Brain Behav Immun Health. (2024) 38:100763. doi: 10.1016/j.bbih.2024.100763, PMID: 38682010 PMC11052898

[55] Longo S Rizza S Federici M. Microbiota-gut-brain axis: relationships among the vagus nerve, gut microbiota, obesity, and diabetes. Acta Diabetol. (2023) 60:1007–17. doi: 10.1007/s00592-023-02088-x, PMID: 37058160 PMC10289935

[56] Rusch JA Layden BT Dugas LR. Signalling cognition: the gut microbiota and hypothalamic-pituitary-adrenal axis. Front Endocrinol (Lausanne). (2023) 14:1130689. doi: 10.3389/fendo.2023.1130689, PMID: 37404311 PMC10316519

[57] Mascia L. Acute lung injury in patients with severe brain injury: a double hit model. Neurocrit Care. (2009) 11:417–26. doi: 10.1007/s12028-009-9242-8, PMID: 19548120

[58] Ott L McClain CJ Gillespie M Young B. Cytokines and metabolic dysfunction after severe head injury. J Neurotrauma. (1994) 11:447–72. doi: 10.1089/neu.1994.11.447, PMID: 7861440

[59] McKeating EG Andrews PJ Signorini DF Mascia L. Transcranial cytokine gradients in patients requiring intensive care after acute brain injury. Br J Anaesth. (1997) 78:520–3. doi: 10.1093/bja/78.5.520, PMID: 9175965

[60] Ziaka M Exadaktylos A. Brain-lung interactions and mechanical ventilation in patients with isolated brain injury. Crit Care. (2021) 25:358. doi: 10.1186/s13054-021-03778-0, PMID: 34645485 PMC8512596

[61] Dickson RP. The microbiome and critical illness. Lancet Respir Med. (2016) 4:59–72. doi: 10.1016/s2213-2600(15)00427-0, PMID: 26700442 PMC4752077

[62] Dickson RP Schultz MJ van der Poll T Schouten LR Falkowski NR Luth JE. Lung microbiota predict clinical outcomes in critically ill patients. Am J Respir Crit Care Med. (2020) 201:555–63. doi: 10.1164/rccm.201907-1487OC, PMID: 31973575 PMC7047465

[63] Guo J Yang L. Regulation effect of the intestinal flora and intervention strategies targeting the intestinal flora in alleviation of pulmonary fibrosis development. Biosci Microbiota Food Health. (2024) 43:293–9. doi: 10.12938/bmfh.2023-100, PMID: 39364128 PMC11444866

[64] Panzer AR Lynch SV Langelier C Christie JD McCauley K Nelson M. Lung microbiota is related to smoking status and to development of acute respiratory distress syndrome in critically ill trauma patients. Am J Respir Crit Care Med. (2018) 197:621–31. doi: 10.1164/rccm.201702-0441OC, PMID: 29035085 PMC6005235

[65] Yagi K Huffnagle GB Lukacs NW Asai N. The lung microbiome during health and disease. Int J Mol Sci. (2021) 22:10872. doi: 10.3390/ijms221910872, PMID: 34639212 PMC8509400

[66] Huffnagle GB Dickson RP Lukacs NW. The respiratory tract microbiome and lung inflammation: a two-way street. Mucosal Immunol. (2017) 10:299–306. doi: 10.1038/mi.2016.108, PMID: 27966551 PMC5765541

[67] Jiang S Li H Zhang L Mu W Zhang Y Chen T. Generic Diagramming Platform (GDP): a comprehensive database of high-quality biomedical graphics. Nucleic Acids Res. (2025) 53:D1670–D1676. doi: 10.1093/nar/gkae973 39470721 PMC11701665

[68] Ma Q Yao C Wu Y Wang H Fan Q Yang Q. Neurological disorders after severe pneumonia are associated with translocation of endogenous bacteria from the lung to the brain. Sci Adv. (2023) 9:eadi0699. doi: 10.1126/sciadv.adi0699, PMID: 37851811 PMC10584344

[69] Cotoia A Paradiso R Ferrara G Borriello G Santoro F Spina I. Modifications of lung microbiota structure in traumatic brain injury ventilated patients according to time and enteral feeding formulas: a prospective randomized study. Crit Care. (2023) 27:244. doi: 10.1186/s13054-023-04531-5, PMID: 37344845 PMC10283314

[70] Bao K Wang M Liu L Zhang D Jin C Zhang J. Jinhong decoction protects sepsis-associated acute lung injury by reducing intestinal bacterial translocation and improving gut microbial homeostasis. Front Pharmacol. (2023) 14:1079482. doi: 10.3389/fphar.2023.1079482, PMID: 37081964 PMC10110981

[71] Zhou X Liao Y. Gut-lung crosstalk in Sepsis-induced acute lung injury. Front Microbiol. (2021) 12:779620. doi: 10.3389/fmicb.2021.779620, PMID: 35003009 PMC8733643

[72] Zhou P Zou Z Wu W Zhang H Wang S Tu X. The gut-lung axis in critical illness: microbiome composition as a predictor of mortality at day 28 in mechanically ventilated patients. BMC Microbiol. (2023) 23:399. doi: 10.1186/s12866-023-03078-3, PMID: 38110878 PMC10726596

[73] Murphy AC Lalor SJ Lynch MA Mills KH. Infiltration of Th1 and Th17 cells and activation of microglia in the CNS during the course of experimental autoimmune encephalomyelitis. Brain Behav Immun. (2010) 24:641–51. doi: 10.1016/j.bbi.2010.01.014, PMID: 20138983

[74] Breit S Kupferberg A Rogler G Hasler G. Vagus nerve as modulator of the brain-gut Axis in psychiatric and inflammatory disorders. Front Psych. (2018) 9:44. doi: 10.3389/fpsyt.2018.00044, PMID: 29593576 PMC5859128

[75] Xia W Li G Pan Z Zhou Q. Hypercapnia attenuates ventilator-induced lung injury through vagus nerve activation. Acta Cir Bras. (2019) 34:e201900902. doi: 10.1590/s0102-865020190090000002, PMID: 31778524 PMC6887097

[76] Li C Chen W Lin F Li W Wang P Liao G. Functional two-way crosstalk between brain and lung: the brain-lung Axis. Cell Mol Neurobiol. (2023) 43:991–1003. doi: 10.1007/s10571-022-01238-z, PMID: 35678887 PMC9178545

[77] Siopi E Galerne M Rivagorda M Saha S Moigneu C Moriceau S. Gut microbiota changes require vagus nerve integrity to promote depressive-like behaviors in mice. Mol Psychiatry. (2023) 28:3002–12. doi: 10.1038/s41380-023-02071-6, PMID: 37131071 PMC10615761

[78] Marano G Mazza M Lisci FM Ciliberto M Traversi G Kotzalidis GD. The microbiota-gut-brain Axis: Psychoneuroimmunological insights. Nutrients. (2023) 15:15. doi: 10.3390/nu15061496, PMID: 36986226 PMC10059722

[79] Ye T Yuan S Kong Y Yang H Wei H Zhang Y. Effect of probiotic Fungi against cognitive impairment in mice via regulation of the fungal microbiota-gut-brain Axis. J Agric Food Chem. (2022) 70:9026–38. doi: 10.1021/acs.jafc.2c03142, PMID: 35833673

[80] Kim SK Guevarra RB Kim YT Kwon J Kim H Cho JH. Role of probiotics in human gut microbiome-associated diseases. J Microbiol Biotechnol. (2019) 29:1335–40. doi: 10.4014/jmb.1906.06064, PMID: 31434172

[81] Eastwood J Walton G Van Hemert S Williams C Lamport D. The effect of probiotics on cognitive function across the human lifespan: a systematic review. Neurosci Biobehav Rev. (2021) 128:311–27. doi: 10.1016/j.neubiorev.2021.06.032, PMID: 34171323

[82] Takada K Shimokawa M Takamori S Shimamatsu S Hirai F Tagawa T. Clinical impact of probiotics on the efficacy of anti-PD-1 monotherapy in patients with nonsmall cell lung cancer: a multicenter retrospective survival analysis study with inverse probability of treatment weighting. Int J Cancer. (2021) 149:473–82. doi: 10.1002/ijc.33557, PMID: 33720422

[83] Lordan C Thapa D Ross RP Cotter PD. Potential for enriching next-generation health-promoting gut bacteria through prebiotics and other dietary components. Gut Microbes. (2020) 11:1–20. doi: 10.1080/19490976.2019.1613124, PMID: 31116628 PMC6973326

[84] Chudzik A Orzyłowska A Rola R Stanisz GJ. Probiotics, prebiotics and Postbiotics on mitigation of depression symptoms: modulation of the brain-gut-microbiome Axis. Biomol Ther. (2021) 11:1000. doi: 10.3390/biom11071000, PMID: 34356624 PMC8301955

[85] Tamtaji OR Taghizadeh M Daneshvar Kakhaki R Kouchaki E Bahmani F Borzabadi S. Clinical and metabolic response to probiotic administration in people with Parkinson's disease: a randomized, double-blind, placebo-controlled trial. Clin Nutr. (2019) 38:1031–5. doi: 10.1016/j.clnu.2018.05.018, PMID: 29891223

[86] Hill C Guarner F Reid G Gibson GR Merenstein DJ Pot B. Expert consensus document. The international scientific Association for Probiotics and Prebiotics consensus statement on the scope and appropriate use of the term probiotic. Nat Rev Gastroenterol Hepatol. (2014) 11:506–14. doi: 10.1038/nrgastro.2014.66, PMID: 24912386

[87] Cunningham M Azcarate-Peril MA Barnard A Benoit V Grimaldi R Guyonnet D. Shaping the future of probiotics and prebiotics. Trends Microbiol. (2021) 29:667–85. doi: 10.1016/j.tim.2021.01.003, PMID: 33551269

[88] Hutkins RW Krumbeck JA Bindels LB Cani PD Fahey G Jr Goh YJ. Prebiotics: why definitions matter. Curr Opin Biotechnol. (2016) 37:1–7. doi: 10.1016/j.copbio.2015.09.001, PMID: 26431716 PMC4744122

[89] Kim CH Park J Kim M. Gut microbiota-derived short-chain fatty acids, T cells, and inflammation. Immune Netw. (2014) 14:277–88. doi: 10.4110/in.2014.14.6.277, PMID: 25550694 PMC4275385

[90] Furusawa Y Obata Y Fukuda S Endo TA Nakato G Takahashi D. Commensal microbe-derived butyrate induces the differentiation of colonic regulatory T cells. Nature. (2013) 504:446–50. doi: 10.1038/nature12721, PMID: 24226770

[91] Maranduba CM De Castro SB de Souza GT Rossato C da Guia FC Valente MA. Intestinal microbiota as modulators of the immune system and neuroimmune system: impact on the host health and homeostasis. J Immunol Res. (2015) 2015:931574. doi: 10.1155/2015/931574, PMID: 25759850 PMC4352473

[92] Wang X Zhang P Zhang X. Probiotics regulate gut microbiota: an effective method to improve immunity. Molecules. (2021) 26:26. doi: 10.3390/molecules26196076, PMID: 34641619 PMC8512487

[93] Strandwitz P. Neurotransmitter modulation by the gut microbiota. Brain Res. (2018) 1693:128–33. doi: 10.1016/j.brainres.2018.03.015, PMID: 29903615 PMC6005194

[94] Xiao J Katsumata N Bernier F Ohno K Yamauchi Y Odamaki T. Probiotic *Bifidobacterium breve* in improving cognitive functions of older adults with suspected mild cognitive impairment: a randomized, double-blind, placebo-controlled trial. J Alzheimers Dis. (2020) 77:139–47. doi: 10.3233/jad-200488, PMID: 32623402 PMC7592675

[95] Srivastav S Neupane S Bhurtel S Katila N Maharjan S Choi H. Probiotics mixture increases butyrate, and subsequently rescues the nigral dopaminergic neurons from MPTP and rotenone-induced neurotoxicity. J Nutr Biochem. (2019) 69:73–86. doi: 10.1016/j.jnutbio.2019.03.021, PMID: 31063918

[96] Ton AMM Campagnaro BP Alves GA Aires R Côco LZ Arpini CM. Oxidative stress and dementia in Alzheimer's patients: effects of Synbiotic supplementation. Oxidative Med Cell Longev. (2020) 2020:2638703. doi: 10.1155/2020/2638703, PMID: 32411323 PMC7201593

[97] Aljumaah MR Bhatia U Roach J Gunstad J Azcarate Peril MA. The gut microbiome, mild cognitive impairment, and probiotics: a randomized clinical trial in middle-aged and older adults. Clin Nutr. (2022) 41:2565–76. doi: 10.1016/j.clnu.2022.09.012, PMID: 36228569

[98] Cammarota G Ianiro G Tilg H Rajilić-Stojanović M Kump P Satokari R. European consensus conference on faecal microbiota transplantation in clinical practice. Gut. (2017) 66:569–80. doi: 10.1136/gutjnl-2016-313017, PMID: 28087657 PMC5529972

[99] Lopetuso LR Deleu S Godny L Petito V Puca P Facciotti F. The first international Rome consensus conference on gut microbiota and faecal microbiota transplantation in inflammatory bowel disease. Gut. (2023) 72:1642–50. doi: 10.1136/gutjnl-2023-329948, PMID: 37339849 PMC10423477

[100] Khoruts A Staley C Sadowsky MJ. Faecal microbiota transplantation for Clostridioides difficile: mechanisms and pharmacology. Nat Rev Gastroenterol Hepatol. (2021) 18:67–80. doi: 10.1038/s41575-020-0350-4, PMID: 32843743

[101] Simpson RC Shanahan ER Scolyer RA Long GV. Towards modulating the gut microbiota to enhance the efficacy of immune-checkpoint inhibitors. Nat Rev Clin Oncol. (2023) 20:697–715. doi: 10.1038/s41571-023-00803-9, PMID: 37488231

[102] Zhao Z Ning J Bao XQ Shang M Ma J Li G. Fecal microbiota transplantation protects rotenone-induced Parkinson's disease mice via suppressing inflammation mediated by the lipopolysaccharide-TLR4 signaling pathway through the microbiota-gut-brain axis. Microbiome. (2021) 9:226. doi: 10.1186/s40168-021-01107-9, PMID: 34784980 PMC8597301

[103] Kim MS Kim Y Choi H Kim W Park S Lee D. Transfer of a healthy microbiota reduces amyloid and tau pathology in an Alzheimer's disease animal model. Gut. (2020) 69:283–94. doi: 10.1136/gutjnl-2018-317431, PMID: 31471351

[104] Li N Dai Z Wang Z Deng Z Zhang J Pu J. Gut microbiota dysbiosis contributes to the development of chronic obstructive pulmonary disease. Respir Res. (2021) 22:274. doi: 10.1186/s12931-021-01872-z, PMID: 34696775 PMC8543848

[105] Feuerstadt P Louie TJ Lashner B Wang EEL Diao L Bryant JA. SER-109, an Oral microbiome therapy for recurrent Clostridioides difficile infection. N Engl J Med. (2022) 386:220–9. doi: 10.1056/NEJMoa2106516, PMID: 35045228

[106] Khanna S Assi M Lee C Yoho D Louie T Knapple W. Efficacy and safety of RBX2660 in PUNCH CD3, a phase III, randomized, double-blind, placebo-controlled trial with a Bayesian primary analysis for the prevention of recurrent Clostridioides difficile infection. Drugs. (2022) 82:1527–38. doi: 10.1007/s40265-022-01797-x, PMID: 36287379 PMC9607700

[107] Chen X Zhang W Lin Z Zheng C Chen S Zhou H. Preliminary evidence for developing safe and efficient fecal microbiota transplantation as potential treatment for aged related cognitive impairments. Front Cell Infect Microbiol. (2023) 13:1103189. doi: 10.3389/fcimb.2023.1103189, PMID: 37113132 PMC10127103

[108] Soveral LF Korczaguin GG Schmidt PS Nunes IS Fernandes C Zárate-Bladés CR. Immunological mechanisms of fecal microbiota transplantation in recurrent Clostridioides difficile infection. World J Gastroenterol. (2022) 28:4762–72. doi: 10.3748/wjg.v28.i33.4762, PMID: 36156924 PMC9476857

[109] Xu C Ban Q Wang W Hou J Jiang Z. Novel nano-encapsulated probiotic agents: encapsulate materials, delivery, and encapsulation systems. J Control Release. (2022) 349:184–205. doi: 10.1016/j.jconrel.2022.06.061, PMID: 35798093

[110] Woo AYM Aguilar Ramos MA Narayan R Richards-Corke KC Wang ML Sandoval-Espinola WJ. Targeting the human gut microbiome with small-molecule inhibitors. Nat Rev Chem. (2023) 7:319–39. doi: 10.1038/s41570-023-00471-4, PMID: 37117817

[111] Eladham MW Selvakumar B Saheb Sharif-Askari N Saheb Sharif-Askari F Ibrahim SM Halwani R. Unraveling the gut-lung axis: exploring complex mechanisms in disease interplay. Heliyon. (2024) 10:e24032. doi: 10.1016/j.heliyon.2024.e24032, PMID: 38268584 PMC10806295

[112] Cao F Jin L Gao Y Ding Y Wen H Qian Z. Artificial-enzymes-armed *Bifidobacterium longum* probiotics for alleviating intestinal inflammation and microbiota dysbiosis. Nat Nanotechnol. (2023) 18:617–27. doi: 10.1038/s41565-023-01346-x, PMID: 36973397

[113] Verdier C Denis S Gasc C Boucinha L Uriot O Delmas D. An Oral FMT capsule as efficient as an Enema for microbiota reconstruction following disruption by antibiotics, as assessed in an *in vitro* human gut model. Microorganisms. (2021) 9:358. doi: 10.3390/microorganisms9020358, PMID: 33670255 PMC7918368

[114] Geng Z Wang X Wu F Cao Z Liu J. Biointerface mineralization generates ultraresistant gut microbes as oral biotherapeutics. Sci Adv. (2023) 9:eade0997. doi: 10.1126/sciadv.ade0997, PMID: 36930714 PMC10022893

[115] Rinott E Youngster I Yaskolka Meir A Tsaban G Zelicha H Kaplan A. Effects of diet-modulated autologous fecal microbiota transplantation on weight regain. Gastroenterology. (2021) 160:158–173.e10. doi: 10.1053/j.gastro.2020.08.041, PMID: 32860791 PMC7755729

[116] Porcari S Benech N Valles-Colomer M Segata N Gasbarrini A Cammarota G. Key determinants of success in fecal microbiota transplantation: from microbiome to clinic. Cell Host Microbe. (2023) 31:712–33. doi: 10.1016/j.chom.2023.03.020, PMID: 37167953

[117] Ianiro G Punčochář M Karcher N Porcari S Armanini F Asnicar F. Variability of strain engraftment and predictability of microbiome composition after fecal microbiota transplantation across different diseases. Nat Med. (2022) 28:1913–23. doi: 10.1038/s41591-022-01964-3, PMID: 36109637 PMC9499858

[118] Park SY Seo GS. Fecal microbiota transplantation: is it safe? Clin Endosc. (2021) 54:157–60. doi: 10.5946/ce.2021.072, PMID: 33827154 PMC8039753

[119] Cammarota G Ianiro G Kelly CR Mullish BH Allegretti JR Kassam Z. International consensus conference on stool banking for faecal microbiota transplantation in clinical practice. Gut. (2019) 68:2111–21. doi: 10.1136/gutjnl-2019-319548, PMID: 31563878 PMC6872442

[120] Danne C Rolhion N Sokol H. Recipient factors in faecal microbiota transplantation: one stool does not fit all. Nat Rev Gastroenterol Hepatol. (2021) 18:503–13. doi: 10.1038/s41575-021-00441-5, PMID: 33907321

[121] Zuurveld M Kiliaan PCJ van Grinsven SEL Folkerts G Garssen J Van't Land B. Ovalbumin-induced epithelial activation directs monocyte-derived dendritic cells to instruct type 2 inflammation in T cells which is differentially modulated by 2'-Fucosyllactose and 3-Fucosyllactose. J Innate Immun. (2023) 15:222–39. doi: 10.1159/000526528, PMID: 36215948 PMC10643896

